# *P*-Chloroaniline Poisoning Causing Methemoglobinemia: A Case Report and Review of the Literature

**DOI:** 10.1155/2015/208732

**Published:** 2015-03-12

**Authors:** Anna Sarah Messmer, Christian Hans Nickel, Dirk Bareiss

**Affiliations:** Department of Emergency Medicine, University Hospital Basel, Petersgraben 2, 4031 Basel, Switzerland

## Abstract

*Background*. Methemoglobin (MetHb) most commonly results from exposure to an oxidizing chemical but may also arise from genetic, dietary, or even idiopathic etiologies. *P*-chloroaniline (PCA) was one of the first substances described in the context of acquired methemoglobinemia. *Case Report*. We report the case of a cyanotic chemistry worker who presented to our emergency department (ED) after working with PCA. His peripheral oxygen saturation (SpO_2_) measured by pulse oximetry was at 81% and remained on that level despite oxygen administration (100% oxygenation via nonrebreather mask). His MetHb level was measured at 42.8% in arterial blood gas analysis. After treatment with intravenous methylene blue cyanosis resolved and the patient was discharged after 36 hours of observation. *Conclusion*. Acquired methemoglobinemia is a treatable condition, which may cause significant morbidity and mortality. The knowledge about the most common causes, fast diagnostic, and proper treatment is crucial.

## 1. Background

Methemoglobinemia is a treatable condition that may cause significant morbidity and mortality [1]. Various systems normally operate to keep methemoglobin (MetHb) at physiologic level, which is less than 1% of the total hemoglobin concentration [2]. Methemoglobinemia is a result of the oxidation of ferrous iron (Fe^2+^) to ferric iron (Fe^3+^) within the hemoglobin molecule.

This becomes important in the context of oxygen transport and delivery: the ferric state is unable to bind oxygen, and the remaining ferrous (reduced) hemoglobin has a higher affinity for oxygen molecules (allosteric effect), shifting the oxygen-hemoglobin dissociation curve to the left. Consequently, the release of oxygen to the tissues is limited [1–4]. As an example, a patient with a hemoglobin level of 10 g/dL who has 50% MetHb has only 5 g/dL functional hemoglobin able to bind oxygen and deliver it to tissues.

Most commonly, methemoglobinemia is acquired and a result of ingestion or skin exposure to an oxidizing agent such as certain medications or chemicals; see Table 1 [1, 2, 5, 6]. Aniline and its derivatives have been one of the first substances described to cause methemoglobinemia. Due to the awareness of its toxicity in industrial products, safety measures have been undertaken and acute aniline poisoning became rare in developed countries, reflected in case reports dated back to several decades [7, 8]. However, our case report shows that accidental aniline or* p*-chloroaniline (PCA) poisoning can nowadays still induce significant, life-threatening methemoglobinemia.

## 2. Case Report

A 56-year-old patient with known epilepsy in his past medical history presented to the emergency department (ED) with generalized cyanosis four hours after cleaning a floor in a chemical factory. He was using PCA in solid form, which was dissolved with methanol. While working, he was using a facemask and a full body protection suit, which, according to his team leader, might have been defective. Later, a work colleague noticed that his skin color turned blue. According to the local occupational health guidelines, the patient immediately changed his clothes and took a shower to decontaminate.

In the ED, he was mildly confused and giddy, but he did not suffer from dyspnea. His vital signs showed mild tachycardia with 107 beats per minute, blood pressure of 156/89 mmHg, and peripheral oxygen saturation (SpO_2_) of 81% on a nonrebreather facemask (100% oxygenation). Arterial blood drawn immediately was of dark brown color and the blood gas analysis revealed a pH of 7.401, pO_2_ of 8.33 kpa (62.5 mmHg), pCO_2_ of 5.49 kpa (41.2 mmHg), and an arterial oxygen saturation (SaO_2_) of 93.4%. The fraction MetHb measured in arterial blood by spectrophotometry was found to be elevated at 42.8%, while the fraction of oxyhemoglobin (O_2_Hb) was 54.6%, and the fraction of reduced hemoglobin (HHb) was 3.9% (see Table 2).

The patient was treated immediately with 150 mg (2 mg/kg) intravenous methylene blue over 5 minutes. A sample of arterial blood gas obtained 30 minutes after treatment revealed a MetHb level of 12.3%; therefore, another dose of 150 mg of methylene blue was given. The patient's condition improved rapidly, the cyanosis resolved, and the patient was transferred to our intensive care unit for observation of possible rebound methemoglobinemia. Serial MetHb measurements showed a continued decrease to below 2%. The patient was discharged 36 hours later.

## 3. Discussion

In 1959, aniline poisoning has been first described in a case series of marking-ink poisoning causing methemoglobinemia in babies [7]. Later, more cases have been published, most often in correlation with dye or herbicides exposure [9–12]. PCA is a colorless or slightly amber colored crystalline solid with a mild aromatic odor. The chemical is soluble in water and in common organic solvents, such as methanol used in our case. The general public may be exposed to PCA from the use of PCA-based dyed or printed textiles, papers, cosmetics, and pharmaceutical products. It can be dermal (wearing of clothes, use of soaps, or mouthwashes), oral (small children sucking clothes and other materials and use of mouthwashes), or direct entry into the bloodstream (e.g., through breakdown products of chlorhexidine in spray antiseptics) [9, 13]. The mechanism of exposure in our case remains unclear. The team leader, who accompanied the patient to the ED, hypothesized a defective protection suit, whereas the PCA could have contaminated the skin.

Patients with methemoglobinemia typically present with skin discoloration (“chocolate cyanosis”), especially of the nails, lips, and ears, whereas the color is more often brown rather than blue. Even at low levels of MetHb, this discoloration can be striking [14].

The characteristic chocolate brown color of the blood is used as a simple bed side test to determine MetHb levels (visible at levels from 15% upwards) to guide treatment in settings of limited resources [15].

Acute methemoglobinemia should be suspected in patients with central cyanosis with low peripheral oxygen saturation not responding to high flow oxygen therapy [16]. The reason is that pulse oximetry may be inaccurate for monitoring oxygen saturation in the presence of methemoglobinemia since conventional pulse oximeters measure ultraviolet absorption at only two wavelengths (940 and 660 nm) to differentiate oxyhemoglobin from deoxyhemoglobin. To determine the oxygen saturation, the oximeter calculates the ratio of absorbance at the 2 wavelengths. A ratio of absorbance (660 nm/940 nm) of 0.43 corresponds to 100%, whereas a ratio of 1.0 corresponds to a saturation of 85%. MetHb absorbs light equally at both 940 and 660 nm. In the presence of 100% MetHb the ratio of absorbance of light at 660 nm over 940 nm is about 1.0. Therefore, at higher MetHb levels, SaO_2_ tends toward 85% regardless of the true percentage of oxyhemoglobin [2, 17]. In our case, the peripheral oxygen saturation was 81%.

Another interesting phenomenon is demonstrated in our case report: the oxygen saturation shown in the arterial blood gas analysis (SaO_2_) may be falsely elevated in patients with methemoglobinemia. The value is calculated based on the patients' pO_2_. The latter refers to dissolved gas and not to oxygen molecules bound to hemoglobin. Subjects with MHb may have normal pO_2_ levels despite life-threatening MHb [2].

Additionally, if an oxygen saturation gap >5% exists between the calculated arterial saturation (SaO_2_) in the blood gas analysis and the reading from the pulse oximeter (SpO_2_) abnormal hemoglobin (e.g., methemoglobinemia) should be suspected [1, 18]. In our case, the gap was 12.4%.

Regardless of etiology, the severity of symptoms depends on the MetHb levels, reported as a percentage of total hemoglobin. Cyanosis caused by MetHb becomes clinically apparent at a MetHb level of about 15% of total hemoglobin. At levels between 30 and 50%, as observed in our patient, dyspnea, headache, weakness and fatigue, dizziness, and sometimes syncope are described. Levels above 70% may cause death [2, 14]. Anemia, acidosis, respiratory compromise, and cardiac disease may make patients more symptomatic than expected for a given MetHb level.

The most effective and widely used antidotal therapy is methylene blue, which was first described in 1933 in aniline dye poisoning [19, 20].

Treatment should be guided by the severity of the disorder and blood levels of MetHb represent a secondary parameter in the definition of the treatment. However, several authors suggest that methylene blue should be considered with MetHb above 30%, regardless of the presence of symptoms [14, 20, 21]. Symptomatic patients or patients with preexisting conditions that interfere with tissue oxygenation should be treated at levels between 10% and 30%. Standard dose of the intravenous administration is 1 to 2 mg/kg given as a 1% solution over 5 minutes [4]. In our case, the MetHb concentration after 30 min was still elevated at 12.3%, and the patient seemed to still be clinically unwell. We were concerned about the patient's state and the fact that he may develop a possible rebound methemoglobinemia. Therefore, we have applied a second dose of methylene blue.

Notably, “desaturation” on the pulse oximeter after methylene blue administration, as seen in our patient, has been well documented and is known to be a result of interference by methylene blue on light absorbance [22]. This interference complicates the assessment of the patient's ability to oxygenate properly and may lead to administration of repeated doses of methylene blue. The patient's clinical status will provide the most useful information about their ability to oxygenate. According to the literature, a repeated dose may be necessary in severe cases of MetHb levels over 60% or if the patient's condition does not improve [20]. The maximal dose should not exceed 7 mg/kg, since methylene blue at high doses may also precipitate MetHb formation [14, 20].

Methylene blue should be used with caution in individuals with severe renal insufficiency and in young patients with G6PD deficiency. This group of patients can develop hemolytic anemia characterized by formation of Heinz bodies without any reduction in MetHb levels [14, 20, 23]. Alternative therapies include exchange transfusion, hyperbaric oxygen, and ascorbic acid [24].

Excretion of PCA in humans occurs primarily via the urine, with PCA and its conjugates appearing as early as 30 min after exposure. Excretion takes place mainly during the first 24 h and is almost complete within 72 h. Nephrotoxic and hepatotoxic potential of PCA has only been described in animal studies [13].

## 4. Conclusion

Our case shows a rare cause of methemoglobinemia. However, methemoglobinemia can be a complication of commonly used or prescribed medication in the ED, that is, nitroglycerin, local anesthetics such as lidocaine, or metoclopramide. Methemoglobinemia should be suspected in a patient presenting with cyanosis not responsive to oxygen administration. The administration of methylene blue should be considered as antidotal therapy for MetHb concentrations over 30%.

## Figures and Tables

**Table 1:** Substances that can cause MetHb^1^.

Drugs		Chemical agents
Acetaminophen	Methylene blue	Acetanilide
Ammonium nitrate	Metoclopramide	Alloxan
Amyl nitrate	Nitrates/nitrites	Aminophenol
Anticonvulsants	Nitric oxide	Anilines
Valproic acid	Nitrofurantoin	Automobile exhaust fumes
Phenytoin	Nitroglycerine	Benzene
Antimalaria drugs	Nitroprusside	Bivalent copper
Chloroquine	Nitrous oxide	Burning wood and plastic
Quinacrine	Isobutyl nitrate	Chlorates
Primaquine	Oral hypoglycemics	Chromates
Bismuth subnitrate	*p*-Aminosalicylic acid	Dimethyl sulfoxide
Dapsone	Phenacetin	Dinitrophenol
Flutamide	Phenazopyridine	Fumes
Hydroxylamine	Piperazine	Naphthalene
Local anesthetics	Rifampin	Nitrates
Benzocaine	Riluzole	Nitrites
Bupivacaine	Sulfadiazine	Nitrobenzene
Lidocaine		Nitroethane
Prilocaine		Nitrophenol
EMLA^*^		Paraquat
		Propanil
		Toluidine
		Trinitrotoluene (TNT)

This list is not complete. ^1^References: see [6, 21, 25]. ^*^Eutectic mixture of local anesthetics.

**Table 2:** Arterial blood gas before and after administration of first dose of methylene blue 150 mg.

	Before methylene blue	After methylene blue
FiO_2_ (fraction of inspired oxygen)	21%	100%
pH	7.401	7.378
pCO_2_ [kPa]	5.59	5.01
pO_2_ [kPa]	8.33	49.6
SaO_2_ [%]	93.4	100
HCO_3_ [mmol/L]	25.5	22.1
Oximetry results		
Hb (hemoglobin [g/L])	130	108
FO_2_Hb (fractional oxyglobin [%])	54.6	90.2
FCOHb (fractional carboxyhemoglobin [%])	0	0
FMetHb (fractional MetHb [%])	42.8	12.3
Lactate [mmol/L]	0.7	0.4

Arterial whole blood was analyzed on an ABL90 series blood gas analyzer, Radiometer Medical, Denmark. Arterial blood gas analyzers are based on electrochemistry and measure pH, pCO_2_, and pO_2_. Serum bicarbonate (HCO_3_) and oxygen saturation (SaO_2_) are calculated values (using the Henderson-Hasselbalch equation for serum bicarbonate and the standard oxygen-hemoglobin saturation curve for oxygen saturation).

## List of Abbreviations

MetHb: Methemoglobin
PCA: * p*-Chloroaniline
ED: Emergency department
SpO_2_: Peripheral oxygen saturation
SaO_2_: Arterial oxygen saturation
pO_2_: Partial pressure of oxygen
pCO_2_: Partial pressure of carbon dioxide.
